# The impact of career plateau on job performance: a cognitive appraisal perspective

**DOI:** 10.3389/fpsyg.2026.1756016

**Published:** 2026-09-08

**Authors:** Xinyu Bao, Yueming Li, Lijun Zhuo

**Affiliations:** 1 School of Public Health and Nursing, Hubei University of Science and Technology, Xianning, China; 2 School of History and Civilization, Shaanxi Normal University, Xi’an, China; 3 School of Health Management, Fujian Medical University, Fuzhou, China; 4 School of Medicine and Health Management of Tongji Medical College, Huazhong University of Science and Technology, Wuhan, China

**Keywords:** career plateau, cognitive appraisal theory of stress, emotional exhaustion, job performance, organizational support

## Abstract

There is little theoretical explanation or robust evidence to support the relationship between career plateau and job performance in physicians. This study adopts the cognitive appraisal theory of stress as a new theoretical framework for the relationships between career plateau, emotional exhaustion, perceived organizational support (POS), and job performance. A survey was conducted among physicians in Chinese public hospitals, yielding 466 valid responses. Hierarchical regression analysis and the PROCESS macro (Model 5) were used to explore mediation and moderation effects using 5,000 bootstrap samples. Both hierarchy plateau and job content plateau are related to poor job performance and are indirectly associated with poor job performance via emotional exhaustion (*p* < 0.001). Additionally, POS can buffer the negative association between job content plateau and job performance (*p* < 0.05) but does not moderate the relationship between hierarchial plateau and job performance (*p* > 0.05). This study provides a novel theoretical explanation of how career plateau is negatively associated with physicians’ job performance and offers practical guidance for hospital managers in managing poor performance and the negative emotions that may trigger it.

## 1 Introduction

Career advancement involves tangible and intangible rewards deemed critical to employees (Lam and Schaubroeck, 2000). Individuals strive for continuous career growth, while the phenomenon of career plateau is often overlooked. Employees may experience two forms of career plateau. A hierarchy plateau occurs when they perceive a low likelihood of future promotion, while a job content plateau arises when their work lacks new responsibilities or challenges (Ference et al., 1977; Milliman, 1992). The majority of studies on career plateau have focused on corporate employees, with limited research among public sector workers, particularly hospital employees (Yang et al., 2018). In China’s hospitals, relatively flat organizational structures restrict upward mobility, increasing the risk of hierarchy plateau. Physicians must balance clinical care with teaching duties. This heavy workload often leaves little time for professional development, contributing to job content plateau.

Both forms of career plateau are often described as stressful experiences linked to various adverse work outcomes, such as reduced job performance (Yang et al., 2018). Previous research has examined these effects through several theoretical lenses, such as the conservation of resources (COR) theory, the social exchange theory, and the social cognitive theory (Ng and Yang, 2023; Yang et al., 2018; Yang et al., 2025; Gustafsson and Kärreman, 2023). However, findings on the relationship between career plateau and job performance remain inconsistent (Allen et al., 1998; Hurst et al., 2017; Hurst et al., 2012; Yang et al., 2025), and emotions have generally been overlooked in career plateau research in favor of work-related outcomes (Ng and Yang, 2023). These gaps suggest the need to explore additional theoretical perspectives that can clarify how and when career plateau influences job performance, as well as how emotional factors shape this relationship.

Cognitive appraisal theory of stress may help us address the aforementioned issues (Folkman et al., 1986). According to this theory, stress reactions are not determined solely by external stressors but depend on how individuals cognitively evaluate these stressors. Although career plateau represents a potentially stressful career condition, its negative consequences depend on employees’ subjective appraisal processes. Problem-focused coping refers to directing attention toward the source of stress and taking proactive steps to reduce its negative impact. Prior studies grounded in resource, social exchange, and self-efficacy theories have largely emphasized this approach. Emotion-focused coping refers to directing attention toward the emotions arising from the problem. When employees perceive stressors as uncontrollable and feel unable to alter external circumstances, they tend to adopt these emotion-focused coping strategies. Therefore, emotional exhaustion may reflect the psychological pathway through which employees’ cognitive evaluation of career plateau is translated into impaired job performance. Globally, physicians often report high levels of emotional exhaustion, defined as feelings of emotional depletion resulting from prolonged work-related stress (Yoon et al., 2010). One study has reported a negative association between hierarchy plateau and emotional exhaustion, while the association between job content plateau and emotional exhaustion remains unclear (Wang et al., 2014).

There is also an increasing need for research that explores whether the negative impact of career plateau can be mitigated or its potential positive outcomes enhanced. The cognitive appraisal theory of stress emphasizes the role of situational factors in shaping how individuals perceive and respond to stress and highlights that individuals’ responses to stress depend not only on their evaluation of the stressor but also on their perceived ability to cope with it. In this process, perceived organizational support (POS) may serve as an important contextual factor shaping employees’ secondary appraisal. Previous research has rarely considered the impact of organizational contexts (Ma et al., 2021). Organizational support is widely recognized as a crucial resource that promotes positive work outcomes (Kurtessis et al., 2017; Shen et al., 2014) and may play a critical role in helping employees cope with career plateau and in influencing their job satisfaction and performance (Yang et al., 2018; Yang et al., 2025).

Grounded in the cognitive appraisal theory of stress, this study aims to examine the mediating role of emotional exhaustion and the moderating role of POS in the relationship between hierarchy and job content plateau and job performance. Our study offers several contributions. First, we introduce a novel theoretical framework for understanding how career plateau affects job performance, emphasizing the emotional processes involved. Second, we provide empirical evidence that both hierarchy and job content plateaus are negatively associated with physicians’ job performance in hospital settings. By identifying these associations, we offer fresh insights into the mechanisms linking career plateau and performance. Third, our findings demonstrate that POS can buffer the negative association between job content plateau and job performance, underscoring the importance of organizational support in helping employees manage career plateau.

## 2 Hypothesis development

### 2.1 Career plateau and job performance

Previous research has shown that both forms of career plateau can be negatively associated with job performance and organizational citizenship behavior (Hurst et al., 2017; Hurst et al., 2012). It has been suggested that employees experiencing a career plateau may exhibit high or low levels of job performance, referred to as “solid citizens” and “deadwood,” respectively (Ference et al., 1977). For physicians, critical resources, such as training and surgical opportunities, are essential for career development but may be limited. Experiencing a career plateau can signal the loss or lack of access to these key resources. As a result, physicians may experience lower job performance. Specifically, those experiencing a hierarchy plateau may face poor working conditions or limited advancement opportunities, while those facing a job content plateau may struggle with outdated knowledge or repetitive tasks, both of which may hinder performance. Prior research has shown that promotion failures can lead to unproductive behaviors (Fine et al., 2016) and emotional distress (Zhu et al., 2022). These effects are often linked to perceptions of denied opportunities, pessimistic interpretations of prospects, and uncertainty about career advancement (Brands and Fernandez-Mateo, 2017; Dlugos and Keller, 2021; Vough and Caza, 2017; Zhu et al., 2022). Accordingly, we hypothesize that both hierarchy and job content plateaus are independently associated with decreased job performance.

*H1a:* Hierarchy plateau is negatively associated with job performance.

*H1b:* Job content plateau is negatively associated with job performance.

### 2.2 The mediator of emotional exhaustion

Emotions are inevitable across almost all career settings, and emotional responses often vary across different career stages (Gustafsson and Kärreman, 2023). However, the role of emotions is generally overlooked in career development research (Ng and Yang, 2023). Emotional exhaustion, defined as the feeling of being emotionally overextended and depleted by one’s work, reflects a state of fatigue and is a key indicator of work-related strain (Maslach et al., 1997). Career plateau, as a form of work-related stress, has been identified as a major contributor to negative emotional and psychological outcomes in various occupational settings, which may result in reduced job performance (Maslach et al., 1997). According to the cognitive appraisal theory of stress, when physicians perceive limited opportunities for promotion (hierarchy plateau) or insufficient opportunities for learning and professional development (job content plateau), they may evaluate these situations as threats to their career goals and future growth. Such threat appraisals may generate negative emotional responses, such as frustration, disappointment, and helplessness, which may accumulate over time and result in emotional exhaustion. Empirical evidence supports this assumption. For example, a study of 188 banking employees in Taiwan found that hierarchy plateau was positively associated with emotional exhaustion and turnover intentions, although job content plateau showed no such relationship (Wang et al., 2014). In contrast, research involving 353 full-time employees in Germany found a positive association between job content plateau and emotional exhaustion (Kao et al., 2022). Prior research has shown that high levels of emotional exhaustion can lead employees to disengage from their work, ultimately resulting in poor job performance (Kao et al., 2022). In general, emotional exhaustion has been associated with increased workplace errors and reduced service quality (Goldberg and Grandey, 2007; Grandey, 2003). Moreover, employees experiencing a career plateau are more likely to engage in counterproductive work behaviors when experiencing negative emotions (Ng and Yang, 2023; Cropanzano et al., 2003). We therefore propose that emotional exhaustion mediates the relationship between career plateau and job performance of physicians.

*H2a:* Emotional exhaustion will mediate the negative relationships between hierarchy plateau and job performance.

*H2b:* Emotional exhaustion will mediate the negative relationships between job content plateau and job performance.

### 2.3 The moderating role of POS

POS reflects how employees view the extent to which their organization values their contributions, as well as how much the organization cares about their well-being (Rhoades and Eisenberger, 2002). According to the cognitive appraisal theory of stress, when employees believe that their organization values their contributions and cares about their well-being, they may perceive greater coping capacity and interpret career plateau as a temporary and manageable challenge rather than an uncontrollable threat. Organizational support, such as recognition and developmental opportunities, may therefore reduce the adverse effects of career plateau. Conversely, when organizational support is perceived as insufficient, employees may interpret career plateau as a signal of organizational neglect, strengthening negative appraisals. A study indicated that POS strongly depends on employees’ attributions concerning the organization’s intent behind their receipt of favorable or unfavorable treatment (Kurtessis et al., 2017). POS may serve as a moderator in addressing job content plateau through career mentoring (Kao et al., 2022). The moderation effect of job content plateau on work-related outcomes is still unclear. Prior research has primarily focused on individual-level moderators, such as career anchors (Wen and Liu, 2015) or psychological contract breaches (Lin et al., 2018). POS is widely recognized as a valuable organizational resource that is associated with positive outcomes such as improved job performance, greater job satisfaction, increased organizational commitment, and lower turnover intention (Shen et al., 2014; Kurtessis et al., 2017). POS, as an important moderator, could strengthen the positive effects of organizational support on work-related outcomes and career-related outcomes (Shen et al., 2014; Kurtessis et al., 2017). Given this finding, POS may serve as a protective factor that buffers the negative association between career plateau and job performance.

*H3a:* POS moderates the relationship between hierarchy plateau and job performance in such a way that it is more negative for lower than for higher levels of POS.

*H3b:* POS moderates the relationship between job content plateau and job performance in such a way that it is more negative for lower than for higher levels of POS.

**Figure 1:**
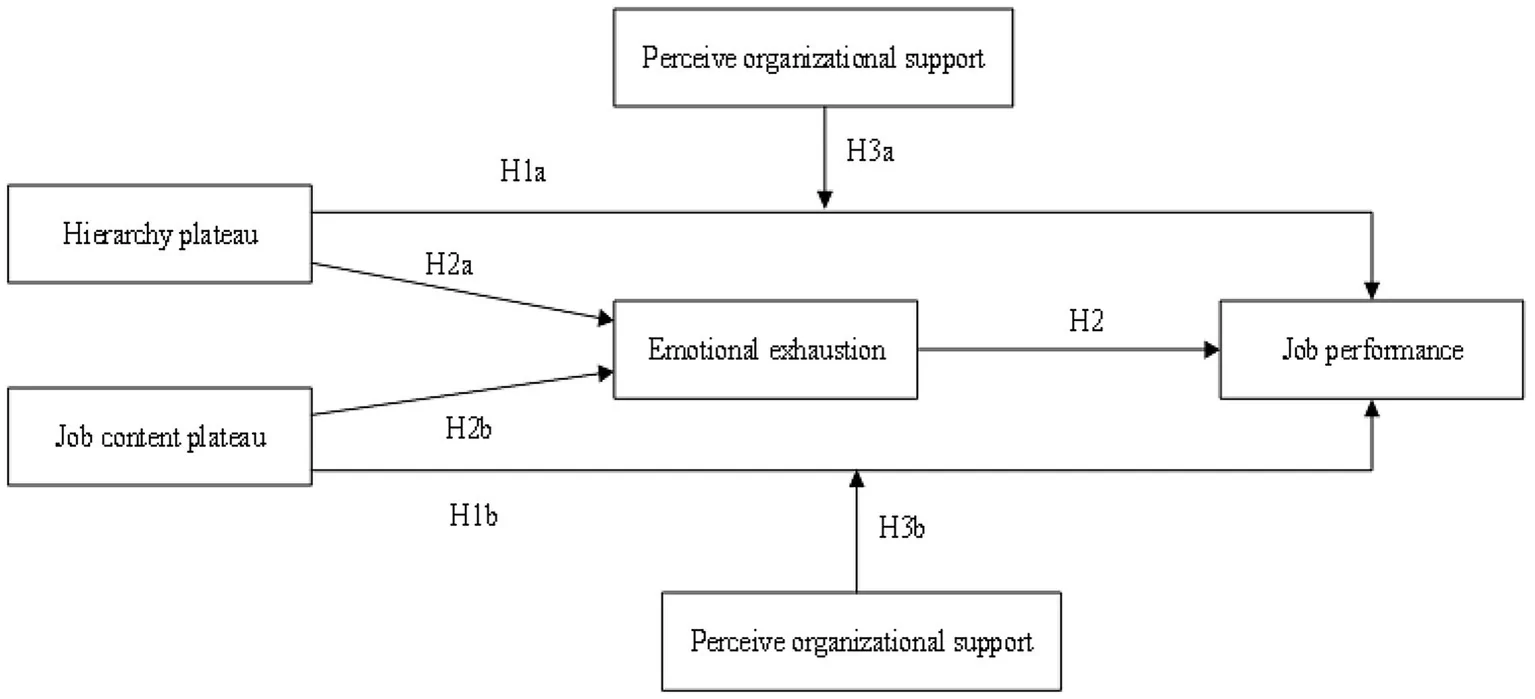
The hypothesis model.

Drawing from the hypotheses, we drew the research model (see Figure 1).

## 3 Materials and methods

### 3.1 Sample and procedure

We collected data from teaching hospitals in Hubei Province, China, through online searches. A multistage sampling design was used to ensure that the study data were representative. In the first stage of sampling, eight public hospitals with the same organizational level were randomly selected in Hubei Province. In the second stage, physicians were selected from each hospital. Of the 580 survey respondents, 466 provided valid responses, resulting in a valid response rate of 80.34%. The inclusion criteria were that participants were physicians who had both teaching and clinical duties. The survey was anonymous. After confirming their informed consent, participants could access the study link or scan the QR code to independently complete the survey questionnaire, thereby ensuring confidentiality.

### 3.2 Measures

Participants responded to each item on a five-point Likert-type scale (1 = “Strongly disagree,” 5 = “Strongly agree”).

*Career plateau* was measured using Milliman’s original 12-item scale (Milliman, 1992), including measures of hierarchy plateau and job content plateau, which is the most commonly used measurement scale (Hu et al., 2022; Yang et al., 2018). Example items are “My opportunities for upward movement are limited in my present organization” and “I expect to be constantly challenged in my job in the future (reversed).” The Cronbach’s alpha coefficients for hierarchy plateau and job content plateau were 0.804 and 0.803, respectively.

*Emotional exhaustion* was measured using Schaufeli’s five-item scale (Schaufeli et al., 1996). An example item is: “I feel used up at the end of the work day.” The Cronbach’s alpha coefficient was 0.891.

*Perceived organizational support* was measured using Eisenberger’s six-item scale (Eisenberger et al., 2001). An example item is: “The hospital cares about my well-being.” The Cronbach’s alpha coefficient was 0.937.

*Job performance* was measured using the Borman and Motowidlo scale, including task performance and contextual performance (Borman and Motowidlo, 1997). An example is: “I can accurately accomplish my work.” The Cronbach’s alpha coefficient was 0.913.

*Control* var*iables*. Gender, marital status, education, work tenure, professional title, and monthly income were control variables in the analysis, as these variables could influence career plateau, emotional exhaustion, and job performance based on previous studies (Hurst et al., 2017; Ng and Yang, 2023; Peng et al., 2022).

### 3.3 Analysis

Before hypothesis testing, a confirmatory factor analysis was performed using AMOS to test the construct validity of the model. Hierarchical regression analysis was used to test the direct and indirect effects of hierarchy and job content plateau on job performance via emotional exhaustion using SPSS 24.0. The moderation analysis was performed using the PROCESS macro (Model 5), with 5,000 bootstrap samples.

## 4 Results

### 4.1 Demographic characteristics of samples

The demographic characteristics of the samples are presented in Table 1. More than half of the participants were male (57.73%), and the average age was 37.63 (SD = 10.14). The majority of participants (82.62%) were married, and 85.62% had a master’s degree or higher. The most common work tenure was 11–20 years (33.91%). The percentage of participants with mid-level professional titles was 49.36%. More than half of the participants earned between CNY¥20,000 and CNY¥30,000 per month. More than one-third of participants worked 41–50 h per week.

**Table 1:** Demography of samples.

Variables	Item	Number (*n*)	Frequency (%)
Sex	Male	269	57.73
	Female	197	42.27
Age	(Mean ± Std) 37.63 ± 6.998		
Marital	Unmarried	72	15.45
	Married	385	82.62
	Other	9	1.93
Education	Bachelor	67	14.38
	Master and above	399	85.62
Work tenure (years)	≤5	124	26.61
	6–10	111	23.82
	11–20	158	33.91
	≥21	73	15.67
Professional title	Junior title	83	17.81
	Intermediate title	230	49.36
	Deputy senior title	153	32.83
Monthly income (RMB 10,000)	≤2	136	29.18
	2–3	272	58.37
	>3	58	12.45
Weekly working hours (hour)	≤40	22	4.72
	41–50	157	33.69
	51–60	147	31.55
	≥61	140	30.04

*N* = 466.

### 4.2 The measurement model

The common method bias is a common systematic error due to artificial covariance caused by data sources, raters, measurement contexts, item context, and characteristics of the items themselves. In this study, the Harman single-factor method was used to assess common method bias validation. The results showed that the eigenvalues of seven factors were all greater than 1, and the total contribution rate was 75.333%. The variance explained by the first principal component was 22.816%, which met the research criteria (less than 50%). Furthermore, the model with five factors (χ2 = 959.163, χ2/df = 2.300, *p* < 0.001, RMSEA = 0.053, GFI = 0.880, CFI = 0.949, TLI = 0.943, SRMR = 0.083) generated a better fit than the model with four factor (χ2 = 3489.847, χ2/df = 8.154, *p* < 0.001, RMSEA = 0.124, GFI = 0.654, CFI = 0.710, TLI = 0.250, SRMR = 0.128) and the model with one factor (χ2 = 7828.512, χ2/df = 18.038, *p* < 0.001, RMSEA = 0.191, GFI = 0.390, CFI = 0.300, TLI = 0.250, SRMR = 0.206). Hence, our results suggest that the common method bias was unlikely to substantially affect the findings.

The discriminant validity results in Table 2 showed that the square root of the average variance extracted (AVE) was greater than the correlation coefficients of each variable, indicating that the variables possess good discriminant validity.

**Table 2:** Descriptive statistics and correlations for study variables.

Variables	Mean	Std	HP	JCP	EE	POS	JP
HP	2.893	0.728	**0.640**				
JCP	2.496	0.636	0.287^**^	**0.669**			
EE	2.764	0.651	0.331^**^	0.347^**^	**0.781**		
POS	3.134	0.991	0.144^**^	0.064	0.006	**0.846**	
JP	3.630	0.797	−0.217^**^	−0.298^**^	−0.264^**^	0.044	**0.751**

*N* = 466 ^*^p < 0.05; ^**^p < 0.01. The bold diagonal elements are the arithmetic square roots of the AVE for this variable. Below the diagonal elements are the correlations between variables. HP, Hierarchy plateau. JCP, Job content plateau. EE, emotional exhaustion. POS, perceived organizational support. JP, Job performance.

### 4.3 Preliminary analyses

Table 2 presents the descriptive statistics and correlations among the independent variables, mediator, moderator, and dependent variable. Hierarchy plateau and job content plateau were positively correlated with emotional exhaustion (r = 0.331, *p* < 0.01; r = 0.347, *p* < 0.01) and negatively correlated with job performance (r = −0.217, *p* < 0.01; r = −0.298, *p* < 0.01), respectively. Emotional exhaustion was negatively correlated with job performance (r = −0.264, *p* < 0.01).

### 4.4 Mediating effect of emotional exhaustion

The variance inflation factor (VIF) of all variables ranged from 1.016 to 2.685, with all values below 5, indicating that multicollinearity was not a concern. The results in Table 3 showed that hierarchy plateau and job content plateau were negatively related to job performance (*p* < 0.001); thus, Hypothesis 1 was supported. Hierarchy plateau was positively related to emotional exhaustion (*β* = 0.336, *p* < 0.001), and emotional exhaustion was negatively related to job performance (β = −0.222, *p* < 0.001). The indirect effect of hierarchy plateau on job performance was −0.082 (95% CI [−0.132, −0.039]). Job content plateau was positively related to emotional exhaustion (β = 0.345, *p* < 0.001), and emotional exhaustion was negatively related to job performance (β = −0.189, *p* < 0.001). The indirect effect of job content plateau on job performance was −0.082 (95% CI [−0.137, −0.034]). Thus, Hypothesis 2 was supported.

**Table 3:** Mediation analysis of hierarchy plateau and job content plateau on job performance, via emotional exhaustion.

Variables	Emotional exhaustion			Job performance				
	Model 1	Model 2	Model 3	Model 4	Model 5	Model 6	Model 7	Model 8
Gender	−0.043	−0.061	−0.046	−0.009	0.003	−0.011	−0.006	−0.015
Marital status	−0.026	−0.016	−0.022	0.030	0.023	0.020	0.026	0.022
Education	−0.025	−0.012	−0.007	0.013	−0.021	−0.024	−0.029	−0.030
Work tenure	−0.079	0.095	0.077	−0.052	0.099	0.121	0.117	0.131
Professional title	0.230	−0.104	−0.072	−0.002	−0.035	−0.058	−0.059	−0.072
Monthly income	0.101	0.000	0.030	0.096	0.013	0.013	−0.008	−0.003
Hierarchy plateau		0.336^***^			−0.224^***^	−0.150^***^		
Job content plateau			0.345^***^				−0.305^***^	−0.240^***^
Emotional exhaustion						−0.222^***^		−0.189^***^
*F*	0.661	8.869^***^	9.515^***^	0.580	3.958^***^	6.370^***^	7.281^***^	8.623^***^
R^2^	0.009	0.119	0.127	0.008	0.057	0.100	0.100	0.131

*N* = 466, ^***^p < 0.001.

### 4.5 Moderating effect of POS

**Figure 2:**
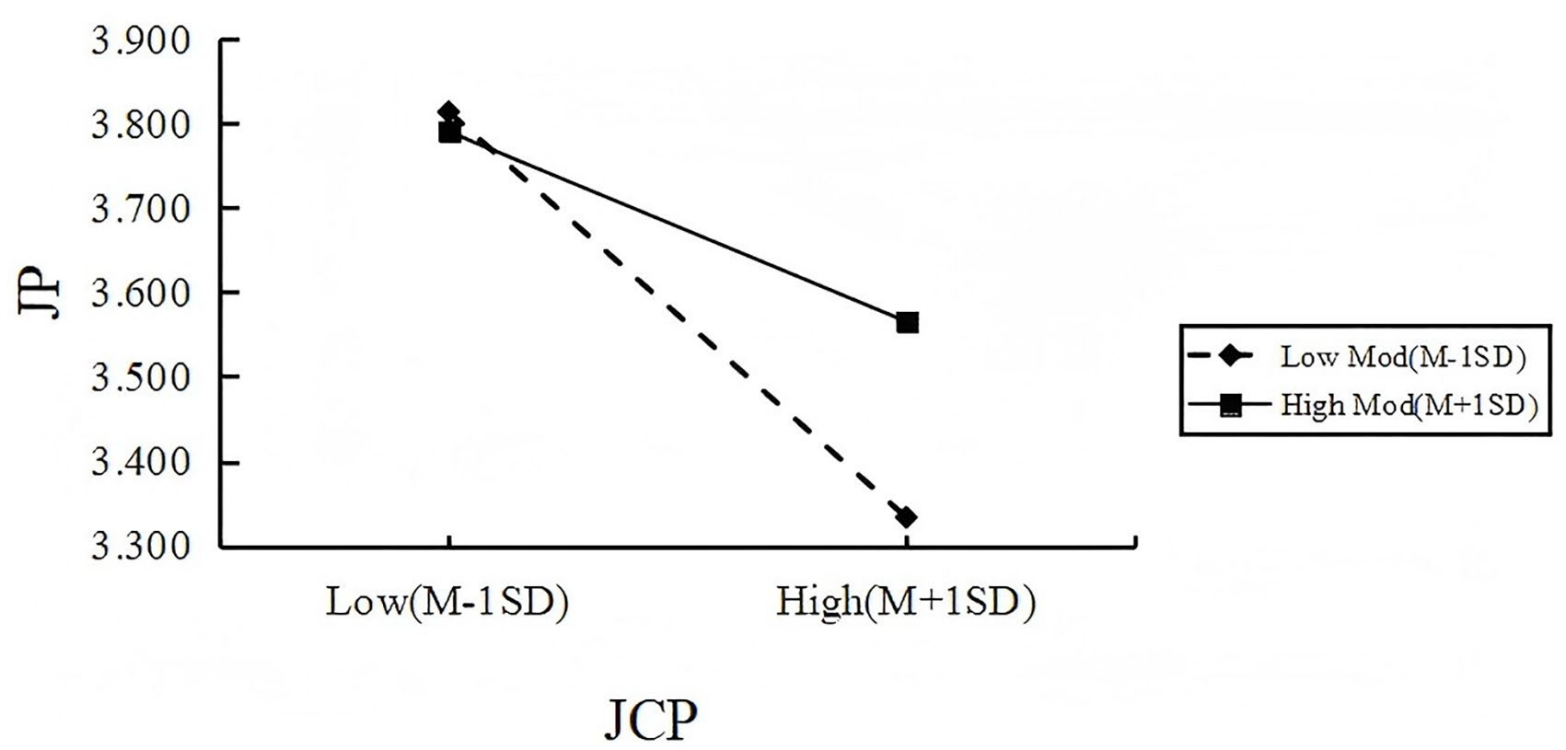
Moderating effect of Perceived organizational support on the relationship between job content plateau and job performance.

In Table 4 and Figure 2, the interaction between job content plateau and POS was significant (estimate = 0.091, 95% CI [0.002, 0.201]), indicating that POS buffered the negative relationship between job content plateau and job performance. In contrast, the interaction between hierarchy plateau and POS was not significant (estimate = 0.083, 95% CI [−0.005, 0.160]). Therefore, Hypothesis 3 was partially supported.

**Table 4:** Moderation analysis of POS in the relationship between hierarchy plateau, job content plateau, and job performance.

Variables	Job performance			
	Model 1	Model 2	Model 3	Model 4
Gender	−0.008	−0.003	−0.013	−0.007
Marital status	0.021	0.017	0.023	0.016
Education	−0.023	−0.024	−0.03	−0.031
Work tenure	0.118	0.113	0.129	0.125
Professional title	−0.059	−0.056	−0.073	−0.072
Monthly income	0.016	0.021	−0.001	0.002
Hierarchy plateau	−0.161^**^	−0.145^**^		
Job content plateau			−0.244^***^	−0.221^***^
Emotional exhaustion	−0.218^***^	−0.221^***^	−0.187^***^	−0.194^***^
POS	0.065	0.062	0.056	0.065
Hierarchy plateau × POS		0.083		
Job content plateau × POS				0.091^*^
*F*	5.907^***^	5.685^***^	7.861^***^	7.522^***^
R^2^	0.104	0.111	0.134	0.142

*N* = 466, ^***^p < 0.001; ^**^p < 0.01; ^*^p < 0.05.

## 5 Discussion

Understanding how career plateau affects emotional exhaustion and job performance is essential for human resource management. Previous research has primarily relied on the COR theory and the social exchange theory to explain the negative outcomes associated with career plateau (Hu et al., 2022; Yang et al., 2018). While existing studies have reported mixed findings on the relationship between career plateau and job performance, this area remains underexplored among physicians (Yang et al., 2018). Emotional factors have been overlooked in this process (Ng and Yang, 2023). Addressing these gaps, our research adopts the cognitive appraisal theory of stress to provide deeper insights into how physicians experience and cope with career plateau.

First, our findings confirm that both hierarchy and job content plateau are directly negatively associated with physicians’ job performance. This contrasts with a previous study conducted in corporate settings, which found that, while hierarchy plateau negatively affected job performance, job content plateau was positively associated (Yang et al., 2025). Unlike many corporate employees, physicians should continuously update their professional skills and medical knowledge. A job content plateau, which limits opportunities for growth and learning, can therefore be negatively associated with their job performance. Physicians experiencing a career plateau may become more vulnerable to stress (Godshalk and Fender, 2015), diverting their energy toward coping with stress, such as focusing on promotion, rather than improving job performance. Moreover, employees who feel stuck in their careers may interpret this stagnation as a sign that the organization values them less, leading to reduced commitment and performance (Godshalk and Fender, 2015). For physicians, this can translate into disengagement from organizational goals, reduced motivation, and diminished job effectiveness.

Second, our study confirms the mediating role of emotional exhaustion in the relationship between both forms of career plateau and job performance. Previous research has explained this relationship through resource depletion, social exchange, or social cognitive theories (Yang et al., 2018); our study adopts a cognitive appraisal perspective. From this view, hierarchy and job content plateaus have a positive effect on emotional responses, which often lead to passive disengagement and, consequently, lower job performance (Zhu et al., 2022). Physicians experiencing a career plateau often feel discouraged and demoralized, as their career goals and aspirations appear unattainable. Those facing a hierarchy plateau may have experienced one or more promotion failures. Previous research has shown that promotion failure is associated with heightened emotional responses, such as anger and reduced self-efficacy, which can lead to emotional exhaustion over time (Zhu et al., 2022; Ng and Yang, 2023). Similarly, physicians facing a job content plateau, marked by routine tasks and limited challenge, may experience boredom and a lack of meaningful engagement. This absence of positive work experiences and psychological resources can also affect emotional exhaustion (Zhu et al., 2022). Our findings suggest that the negative emotions triggered by career plateau contribute to emotional exhaustion, which may, in turn, undermine job performance. However, unlike in other professions, physicians are unlikely to respond to these emotional challenges with overtly destructive behaviors due to the potential consequences for patient safety and care quality. Instead, their response is more likely to manifest as passive disengagement and reduced effectiveness, which nonetheless negatively impacts performance.

Third, our findings revealed that POS buffered the negative effect of job content plateau on job performance, although the interaction coefficient was small, whereas it did not buffer the negative effect of hierarchy plateau. This finding can be further understood from the perspective of the cognitive appraisal theory of stress. According to this theory, employees’ responses to stressors depend not only on the presence of stressors but also on how they evaluate the meaning and controllability of these stressors. Job content plateau primarily reflects employees’ perceptions that their current work lacks challenge, learning opportunities, and professional development. Therefore, when physicians perceive strong organizational support, such support may influence their secondary appraisal by enhancing their perceived coping capacity. Specifically, organizational recognition, professional training, mentoring, and developmental opportunities can help employees reinterpret limited job enrichment as a temporary and manageable situation rather than an irreversible career threat. As a result, POS may reduce negative performance caused by a job content plateau. In contrast, POS did not mitigate the negative relationship between hierarchy plateau and job performance. The core issue of hierarchy plateau lies in the lack of advancement opportunities, which are often constrained by an organization’s structural hierarchy. From a cognitive appraisal perspective, hierarchy plateau represents a perceived limitation in future promotion opportunities, which is closely related to employees’ evaluation of their long-term career prospects and organizational advancement structure. Although organizational support may provide emotional reassurance and coping resources, it may not fundamentally change employees’ appraisal that upward mobility is restricted when promotion opportunities are objectively limited. Thus, employees experiencing a hierarchy plateau may continue to perceive a lack of career progression as an uncontrollable threat, resulting in persistent negative emotions and reduced performance. This distinction suggests that the effectiveness of organizational support depends on the nature of the stressor being appraised. POS is more effective when employees can reinterpret and cope with the stressor but may not be effective when the stressor involves structural constraints that cannot be easily altered. Future research should further investigate other organizational resources (e.g., career development support) that may better explain variations in the effects of different dimensions of career plateau on job performance.

### 5.1 Theoretical contributions

Our study makes several theoretical contributions. First, it reinforces the assumption that career plateau is linked to negative organizational outcomes. Specifically, we demonstrate that both hierarchy and job content plateaus are negatively associated with physicians’ job performance. Second, our research offers a new explanation for why career plateau is related to poor performance. Moving beyond traditional frameworks, we highlight the mediating role of emotional exhaustion through the lens of cognitive appraisal (Hu et al., 2022). While earlier studies have largely overlooked emotional factors in this context, our findings reveal that employees facing a career plateau may rely on emotion-focused coping strategies, which can exacerbate stress and hinder performance (Ng and Yang, 2023). This mediating effect provides new insight into the psychological mechanisms connecting career plateau to job performance. Third, our study demonstrates that high POS can buffer the negative effects of job content plateau on job performance. Our findings emphasize the importance of POS in helping physicians sustain performance when facing a job content plateau, although this effect does not extend to hierarchy plateau.

### 5.2 Practical implications

First, interventions should target both hierarchy and job content plateaus. Hospitals can improve communication around career development pathways to help physicians understand advancement opportunities. To reduce hierarchy plateau, they might establish sub-specialty or disease-specific project teams with leadership roles and offer diverse forms of recognition beyond formal promotions (Bardwick, 1986). Addressing job content plateau requires providing challenging and enriching job assignments. Ensuring strong person-job and person-organization fit can also enhance physicians’ sense of career calling and improve work-related outcomes (Xu et al., 2023).

Second, our study indicates that both hierarchy and job content plateaus increase emotional exhaustion, which in turn negatively affects job performance. Hospital leaders should recognize the emotional challenges physicians face during career plateaus. Incorporating emotional regulation training and promoting positive thinking through human resources development programs is essential. Practices such as mindfulness and regular physical exercise have been shown to enhance emotional regulation, helping physicians manage stress and maintain professional performance (Zhang et al., 2019).

Third, organizational support is critical in influencing physicians’ secondary appraisal by enhancing their perceived coping capacity. Hospitals should actively address physicians’ core needs, such as wellbeing, trust, recognition, career development, mentoring, and training opportunities (Yang et al., 2018). Offering regular professional development programs not only strengthens clinical skills but also motivates physicians to adopt new technologies and practices, enriching their overall work experience.

### 5.3 Limitations and future research recommendations

There are several limitations. First, our study used subjective performance measured by the Borman and Motowidlo scale (Borman and Motowidlo, 1997). Although self-rated performance can capture physicians’ perceptions of their own work effectiveness and professional contributions, reliance on self-reported measures and a cross-sectional design may introduce potential common method bias and limit causal inference. Future research could address these limitations by incorporating multi-source data (e.g., supervisor evaluations or objective organizational records) and adopting longitudinal or experimental designs to further examine the temporal and causal relationships among these variables. Second, our findings confirm that physicians experience both hierarchy and job content plateaus. Career plateau was measured using Milliman’s scale (Milliman, 1992), including two primary dimensions. We suggest that different forms of career plateau may be associated with specific physician characteristics, and future research should explore how other dimensions of career plateau influence job performance in healthcare settings. Third, our data were collected from teaching hospitals in Hubei Province, China, which may limit the generalizability of our findings to other regions, types of healthcare institutions, or non-academic physicians. Given that physicians’ career experiences may vary across healthcare systems, organizational contexts, and professional environments, future research should recruit more diverse samples from different geographic regions, hospital types (e.g., primary care institutions, community hospitals, and rural hospitals), and physicians with varied educational backgrounds to further validate and extend our findings.

## 6 Conclusion

The study proposed a novel theoretical model to explore the relationship between career plateau, emotional exhaustion, POS, and job performance from the perspective of cognitive appraisal theory of stress. Our findings support the model, showing that both hierarchy and job content plateaus have a direct negative impact on job performance, as well as an indirect effect mediated by emotional exhaustion. Furthermore, the results indicate that high POS serves as a protective factor, mitigating the negative relationship between job content plateau and job performance, while it does not buffer the negative relationship between hierarchy plateau and job performance. Hospitals should proactively identify and support physicians experiencing different kinds of career plateau to reduce emotional exhaustion and promote sustained job performance.

## Data Availability

The raw data supporting the conclusions of this article will be made available by the authors, without undue reservation.
